# Crystal structure of 2,2′-oxybis(4-methylquinoline)

**DOI:** 10.1107/S2056989015000717

**Published:** 2015-01-17

**Authors:** Anaelle Tilborg

**Affiliations:** a Department of Chemistry, University of Namur, 61, Rue de Bruxelles, B-5000 Namur, Belgium

**Keywords:** crystal structure, quinoline, benserazide, therapeutic compounds, Parkinson’s disease

## Abstract

The title compound, C_20_H_16_N_2_O, (I), has been unwittingly obtained from the slow evaporation of a saturated solution of commercial benserazide hydro­chloride [benserazide, (II), being one of the principal therapeutic compounds used for the management of Parkinson’s disease, mostly used in combination with levodopa]. The mol­ecule of (I) is composed of two planar 4-methyl­quinoline aromatic moieties [maximum deviations of 0.0104 (18) and 0.016 (2) Å], almost perpendicular to each other [dihedral angle = 89.5 (2)°], bridged by an O atom. The supra­molecular organization consists of a π-bonded chain, resulting from the stacking of mol­ecules related by inversion centres located along direction [111].

## Abstract 2

The title compound was unwittingly obtained from the slow evaporation of a saturated solution of commercial benserazide hydro­chloride. The mol­ecule is composed of two planar 4-methyl­quinoline aromatic moieties, almost perpendicular to each other, bridged by an O atom. The supra­molecular organization consists of a π-bonded chain.

## Chemical context   

Parkinson’s disease is a degenerative disorder of the central nervous system, resulting from the death of dopamine-generating cells, mostly located in the mid-brain. The most obvious symptoms are movement-related: uncontrolled shaking, rigidity, slowness of movement and difficulty in walking. However, behavioral problems and psychiatric depression may also arise (Samii *et al.*, 2004 ▸). Symptomatic treatment of Parkinson’s disease includes daily dopamine administration, principally through l-DOPA (or levodopa) or carbidopa (both being precursors of dopamine) brain metabolization.
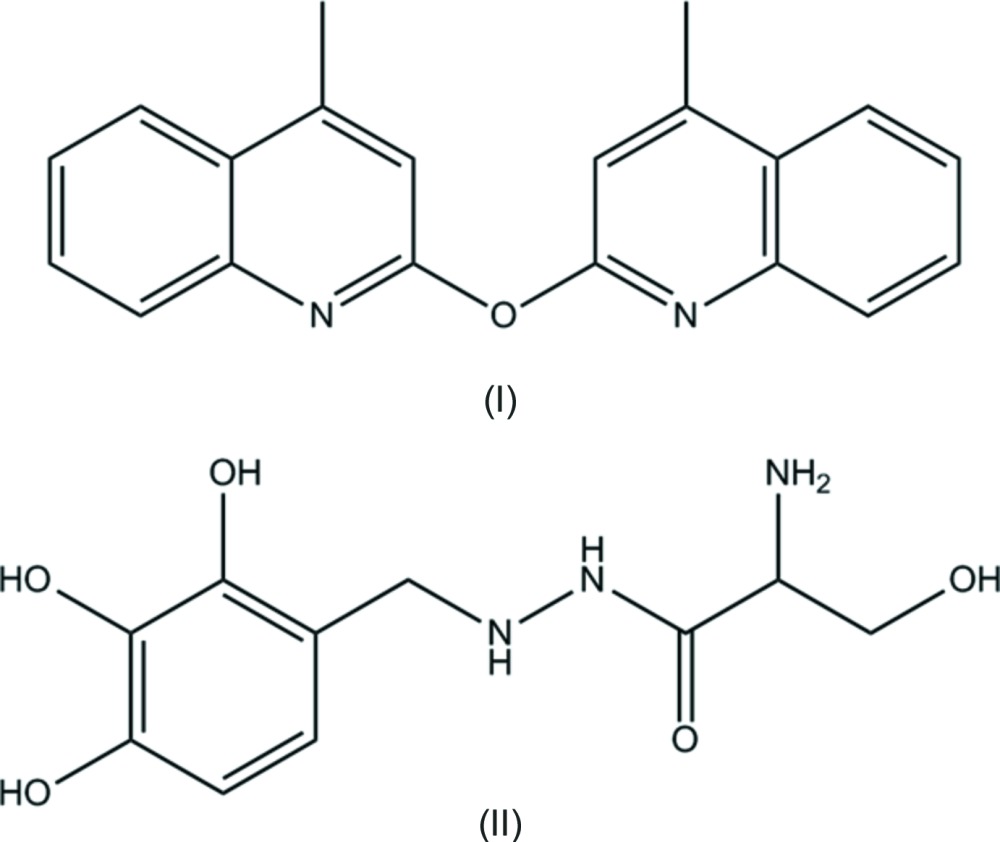


Benserazide [also called Serazide or Ro–4–4602, (II) in the Scheme] is an aromatic l-amino acid deca­rboxylase inhibitor and a DOPA deca­rboxylase inhibitor unable to cross the blood–brain barrier. It is used in combination with levodopa for the symptomatic management of Parkinson’s disease (Clark *et al.*, 1973 ▸; Campanella & Pennetta, 1974 ▸; Bortolanza *et al.*, 2015 ▸).

As benserazide is always administered in combination therapy, it appeared to be a good candidate to search for a solid-state crystalline phase involving it with another thera­peutic mol­ecule, also active in the treatment of Parkinson’s disease. However, little information could be retrieved on the structural aspects of benserazide and, as a first step, recrystallization attempts of the mol­ecule alone have been launched. These crystallization assays have been so far fruitless, but resulted instead in the unwitting obtention of a new mol­ecule, 2,2′-oxybis(4-methylquinoline) (I) with formula C_20_H_16_ON_2_, which is reported herein.

## Structural commentary   

**Figure 1:**
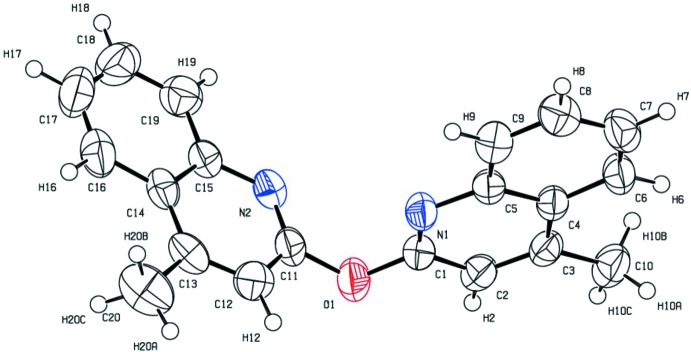
The mol­ecular structure and atom numbering of the title compound. Displacement ellipsoids for the non-H atoms are drawn at the 50% probability level.

The geometry of (I) is fairly predictable, with all bond lengths and valence angles being in the expected range for organic compounds (Allen *et al.*, 1987 ▸). The mol­ecule consists of two planar 4-methyl­quinoline aromatic moieties [the maximum deviations from the mean plane are 0.0104 (18) Å for C1 in the N1,C1–C9 moiety and 0.016 (2) Å for C13 in the N2,C11–C19 unit], almost perpendicular to each other [dihedral angle = 89.5 (2)°] and bound by an oxygen atom which forms an ether link (Fig. 1 ▸).

## Supra­molecular features   

**Figure 2:**
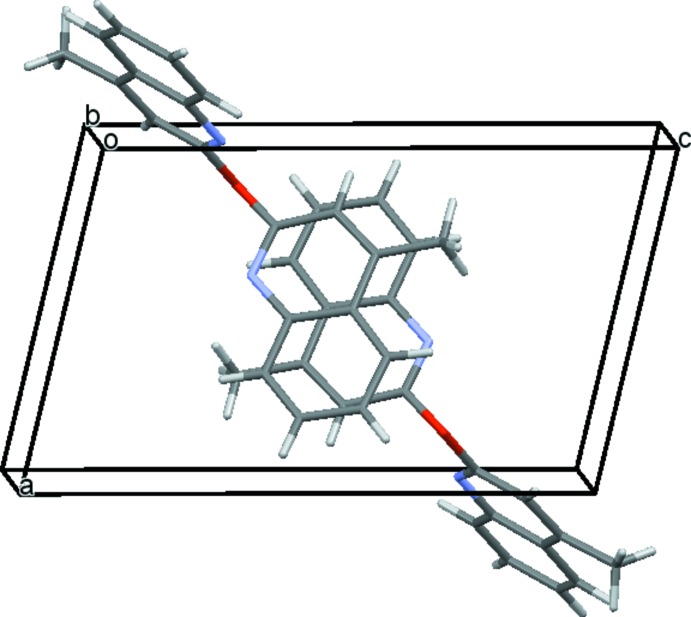
Packing diagram showing one of the π–π inter­actions, stacked around (½, ½, ½).

**Figure 3:**
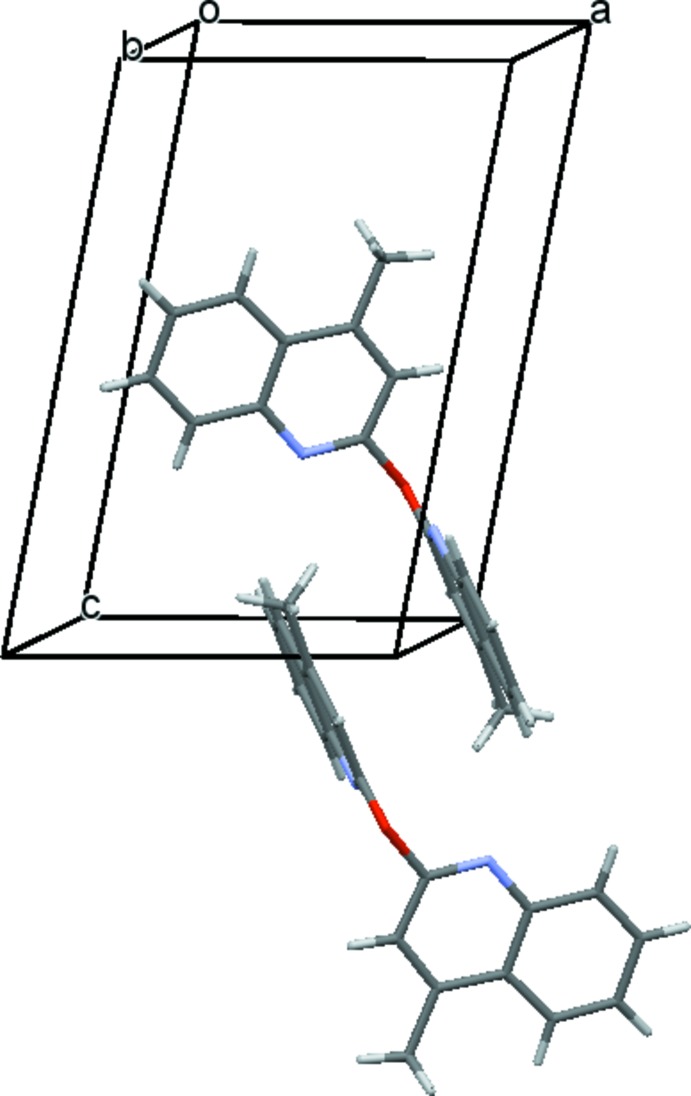
Packing diagram showing the second type of π–π inter­action, stacked around (1, 1, 1).

**Figure 4:**
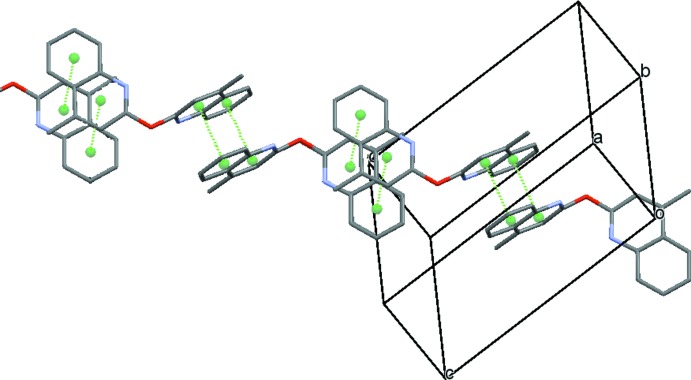
The [111] chain resulting from the two types of π–π inter­actions.

The crystal packing organization is essentially the result of two different types of π-stacking inter­actions involving inversion-related mol­ecules. Table 1 ▸ gives a survey of these π–π stacking inter­actions, in one case around (½, ½, ½) (Fig. 2 ▸) and in the other case around (0, 0, 0);(1, 1, 1) (Fig. 3 ▸). The overall effect of these inter­actions is the formation of chains parallel to [111] (Fig. 4 ▸). As expected from the lack of efficient hydrogen-bond donors, no significant hydrogen bonds linking the chains are present in the structure, as a result of which their mutual inter­action is rather weak.

## Database survey   

**Figure 5:**
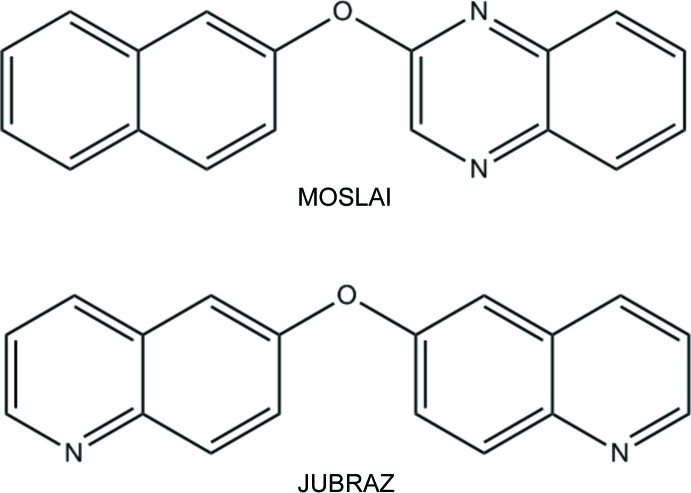
Two similar structures in the CSD [refcodes MOSLAI (Hassan *et al.*, 2009 ▸) and JUBRAZ (Liu *et al.*, 1992 ▸)].

A systematic research in the Cambridge Structural Database (CSD; Version 5.35, update November 2014; Groom & Allen, 2014 ▸) using *ConQuest* (Bruno *et al.*, (2002 ▸) revealed some structures fairly similar to (I), which are presented in Fig. 5 ▸ and identified by their CSD refcodes: MOSLAI (Hassan *et al.*, 2009 ▸) and JUBRAZ (Liu *et al.*, 1992 ▸), the main difference residing in the number and relative position of the nitro­gen atoms in the aromatic rings.

## Synthesis and crystallization   

Prismatic colourless crystals of 2,2′-oxybis(4-methylquinoline) were grown from a 2 ml aqueous saturated solution of benserazide hydro­chloride (purchased from Sigma-Aldrich, Steinheim, Germany; purity level claimed > 98%) (9.3 mg) that was allowed to evaporate slowly at room temperature over 7 days.

Several trials of slow evaporation of aqueous solutions under different temperature conditions (from 277 to 313 K) provided in all cases the same crystals, with the same unit-cell parameters. The main assumption is that the benserazide hydro­chloride has undergone a fundamental structure transformation during the aqueous recrystallization assays, but work is in progress to understand the mechanism, which does not seem to be obvious. Compound (I) could also be a by-product coming from a earlier step in the benserazide synthesis process (even if the qu­antity of crystalline material retrieved is relatively important). A calorimetric study has been undertaken on the crystalline material, and differential scanning calorimetry (DSC) provides an onset temperature (considered as the melting point) of 419.3 K, with no significant endo- or exothermic event before the fusion point. No spontaneous recrystallization occurs when the melt is allowed to cool down.

## Refinement   

Crystal data, data collection and structure refinement details are summarized in Table 2 ▸. The methyl H atoms were located from difference Fourier maps and their positions refined freely. All other H atoms were placed at idealized positions and allowed to ride on their parent atoms, with C—H distances of 0.93 Å and with *U*_iso_(H) = 1.2*U*_eq_(C).

## Supplementary Material

Crystal structure: contains datablock(s) I, Global. DOI: 10.1107/S2056989015000717/bg2545sup1.cif

Structure factors: contains datablock(s) I. DOI: 10.1107/S2056989015000717/bg2545Isup2.hkl

Supporting information file. DOI: 10.1107/S2056989015000717/bg2545Isup3.cml

CCDC reference: 1034614

Additional supporting information:  crystallographic information; 3D view; checkCIF report

## Figures and Tables

**Table 1:** stacking interactions (, ) *Cg*1, *Cg*2, *Cg*3 and *Cg*4 are the centroids of the N1/C1C5, N2/C11C15, C4C9 and C14C19 rings, respectively. *CgCg* is the intercentroid distance, the dihedral angle is between the ring planes and mpd is the mean perpendicular distance between a centroid and the opposite plane.

	*Cg* *Cg*	dihedral angle	mpd
*Cg*1*Cg*1^i^	3.7849(11)	0	3.4446(7)
*Cg*1*Cg*3^i^	3.7775(11)	0.83(8)	3.4345(10)
*Cg*2*Cg*2^ii^	3.6036(11)	0	3.4395(7)
*Cg*2*Cg*4^ii^	3.8817(12)	0.73(10)	3.4462(19)

Symmetry codes: (i) *x*, *y*, *z*; (ii) 1*x*, 1*y*, 1*z*.

**Table 2:** Experimental details

Crystal data	
Chemical formula	C_20_H_16_N_2_O
*M*_r_	300.35
Crystal system, space group	Triclinic, *P* 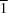
Temperature (K)	293
*a*, *b*, *c* ()	7.8858(5), 7.9226(8), 13.0182(13)
, , ()	104.267(9), 103.576(7), 91.967(7)
*V* (^3^)	762.54(13)
*Z*	2
Radiation type	Mo *K*
(mm^1^)	0.08
Crystal size (mm)	0.5 0.35 0.25

Data collection	
Diffractometer	Oxford Diffraction Xcalibur (Ruby, Gemini) ultra
Absorption correction	Multi-scan (*CrysAlis PRO*; Oxford Diffraction, 2006 ▸)
*T*_min_, *T*_max_	0.960, 1.000
No. of measured, independent and observed [*I* > 2(*I*)] reflections	3234, 2207, 1707
*R*_int_	0.024
(sin /)_max_ (^1^)	0.595

Refinement	
*R*[*F* ^2^ > 2(*F* ^2^)], *wR*(*F* ^2^), *S*	0.046, 0.133, 1.03
No. of reflections	2256
No. of parameters	232
No. of restraints	6
H-atom treatment	H atoms treated by a mixture of independent and constrained refinement
_max_, _min_ (e ^3^)	0.18, 0.14

Computer programs: *CrysAlis PRO* (Oxford Diffraction, 2006 ▸), *SIR92* (Altomare *et al.*, 1994 ▸), *SHELXL97* (Sheldrick, 2015 ▸), *ORTEPIII* (Burnett Johnson, 1996 ▸), *PLATON* (Spek, 2009 ▸) and *Mercury* (Macrae *et al.*, 2006 ▸).

## supplementary crystallographic information

### Crystal data

**Table:**

C_20_H_16_N_2_O	*Z* = 2
*M**_r_* = 300.35	*F*(000) = 316
Triclinic, *P*1	*D*_x_ = 1.308 Mg m^−^^3^
Hall symbol: -P 1	Mo *K*α radiation, λ = 0.71073 Å
*a* = 7.8858 (5) Å	Cell parameters from 1411 reflections
*b* = 7.9226 (8) Å	θ = 3.7–28.7°
*c* = 13.0182 (13) Å	µ = 0.08 mm^−^^1^
α = 104.267 (9)°	*T* = 293 K
β = 103.576 (7)°	Prism, colourless
γ = 91.967 (7)°	0.5 × 0.35 × 0.25 mm
*V* = 762.54 (13) Å^3^	

### Data collection

**Table:**

Oxford Diffraction Xcalibur (Ruby, Gemini) ultra diffractometer	2207 independent reflections
Radiation source: fine-focus sealed tube	1707 reflections with *I* > 2σ(*I*)
Graphite monochromator	*R*_int_ = 0.024
Detector resolution: 10.3712 pixels mm^-1^	θ_max_ = 25.0°, θ_min_ = 3.5°
ω scans	*h* = −7→9
Absorption correction: multi-scan (*CrysAlis PRO*; Oxford Diffraction, 2006)	*k* = −9→5
*T*_min_ = 0.960, *T*_max_ = 1.000	*l* = −14→15
3234 measured reflections	

### Refinement

**Table:**

Refinement on *F*^2^	Primary atom site location: structure-invariant direct methods
Least-squares matrix: full	Secondary atom site location: difference Fourier map
*R*[*F*^2^ > 2σ(*F*^2^)] = 0.046	Hydrogen site location: inferred from neighbouring sites
*wR*(*F*^2^) = 0.133	H atoms treated by a mixture of independent and constrained refinement
*S* = 1.03	*w* = 1/[σ^2^(*F*_o_^2^) + (0.0652*P*)^2^ + 0.0421*P*] where *P* = (*F*_o_^2^ + 2*F*_c_^2^)/3
2256 reflections	(Δ/σ)_max_ < 0.001
232 parameters	Δρ_max_ = 0.18 e Å^−^^3^
6 restraints	Δρ_min_ = −0.14 e Å^−^^3^
0 constraints	

### Special details

**Table:**

Experimental. Absorption correction: CrysAlis PRO, Empirical absorption correction using spherical harmonics, implemented in SCALE3 ABSPACK scaling algorithm.
Geometry. All e.s.d.'s (except the e.s.d. in the dihedral angle between two l.s. planes) are estimated using the full covariance matrix. The cell e.s.d.'s are taken into account individually in the estimation of e.s.d.'s in distances, angles and torsion angles; correlations between e.s.d.'s in cell parameters are only used when they are defined by crystal symmetry. An approximate (isotropic) treatment of cell e.s.d.'s is used for estimating e.s.d.'s involving l.s. planes.
Refinement. Refinement of *F*^2^ against ALL reflections. The weighted *R*-factor *wR* and goodness of fit *S* are based on *F*^2^, conventional *R*-factors *R* are based on *F*, with *F* set to zero for negative *F*^2^. The threshold expression of *F*^2^ > σ(*F*^2^) is used only for calculating *R*-factors(gt) *etc*. and is not relevant to the choice of reflections for refinement. *R*-factors based on *F*^2^ are statistically about twice as large as those based on *F*, and *R*- factors based on ALL data will be even larger.

### Fractional atomic coordinates and isotropic or equivalent isotropic displacement parameters (Å^2^)

**Table:**

	*x*	*y*	*z*	*U*_iso_*/*U*_eq_	
O1	0.13084 (17)	0.38253 (18)	0.26211 (12)	0.0696 (5)	
N1	−0.01449 (17)	0.1056 (2)	0.20704 (12)	0.0470 (4)	
N2	0.38771 (19)	0.2735 (2)	0.32211 (12)	0.0530 (4)	
C1	0.0147 (2)	0.2537 (2)	0.18566 (15)	0.0482 (5)	
C2	−0.0638 (2)	0.2993 (3)	0.08862 (16)	0.0507 (5)	
C3	−0.1801 (2)	0.1811 (3)	0.00729 (15)	0.0458 (5)	
C4	−0.2184 (2)	0.0150 (2)	0.02535 (13)	0.0415 (4)	
C5	−0.1336 (2)	−0.0165 (2)	0.12652 (14)	0.0413 (4)	
C6	−0.3353 (2)	−0.1194 (3)	−0.05297 (16)	0.0535 (5)	
C7	−0.3664 (2)	−0.2761 (3)	−0.03220 (18)	0.0626 (6)	
C8	−0.2839 (2)	−0.3060 (3)	0.06810 (18)	0.0599 (6)	
C9	−0.1692 (2)	−0.1786 (3)	0.14565 (16)	0.0528 (5)	
C10	−0.2614 (3)	0.2203 (4)	−0.09923 (19)	0.0620 (6)	
C11	0.2483 (2)	0.3332 (2)	0.34593 (15)	0.0506 (5)	
C12	0.2112 (2)	0.3641 (3)	0.44746 (18)	0.0560 (5)	
C13	0.3299 (3)	0.3308 (2)	0.53294 (16)	0.0535 (5)	
C14	0.4866 (2)	0.2617 (2)	0.51214 (15)	0.0479 (5)	
C15	0.5100 (2)	0.2357 (2)	0.40549 (15)	0.0472 (5)	
C16	0.6200 (3)	0.2177 (3)	0.59128 (18)	0.0655 (6)	
C17	0.7657 (3)	0.1528 (3)	0.5655 (2)	0.0808 (8)	
C18	0.7892 (3)	0.1314 (3)	0.4606 (2)	0.0797 (7)	
C19	0.6640 (3)	0.1713 (3)	0.38176 (19)	0.0644 (6)	
C20	0.2972 (4)	0.3663 (4)	0.6447 (2)	0.0863 (8)	
H2	−0.0360	0.4095	0.0804	0.061*	
H6	−0.3920	−0.1009	−0.1199	0.064*	
H7	−0.4432	−0.3639	−0.0852	0.075*	
H8	−0.3071	−0.4128	0.0821	0.072*	
H9	−0.1140	−0.1998	0.2120	0.063*	
H10A	−0.386 (3)	0.211 (3)	−0.1129 (17)	0.070 (6)*	
H10B	−0.229 (3)	0.137 (3)	−0.159 (2)	0.081 (7)*	
H10C	−0.219 (3)	0.337 (4)	−0.100 (2)	0.096 (8)*	
H12	0.1062	0.4072	0.4572	0.067*	
H16	0.6078	0.2334	0.6623	0.079*	
H17	0.8511	0.1222	0.6186	0.097*	
H18	0.8912	0.0895	0.4446	0.096*	
H19	0.6801	0.1560	0.3116	0.077*	
H20A	0.186 (2)	0.408 (3)	0.648 (3)	0.137 (12)*	
H20B	0.306 (3)	0.259 (3)	0.669 (3)	0.140 (12)*	
H20C	0.389 (3)	0.451 (3)	0.698 (2)	0.129 (11)*	

### Atomic displacement parameters (Å^2^)

**Table:**

	*U*^11^	*U*^22^	*U*^33^	*U*^12^	*U*^13^	*U*^23^
O1	0.0706 (9)	0.0484 (9)	0.0657 (10)	−0.0002 (6)	−0.0204 (7)	0.0072 (7)
N1	0.0449 (9)	0.0537 (10)	0.0366 (9)	0.0033 (7)	0.0035 (6)	0.0079 (8)
N2	0.0590 (11)	0.0537 (10)	0.0368 (9)	0.0031 (8)	0.0042 (7)	0.0023 (8)
C1	0.0407 (10)	0.0487 (11)	0.0465 (12)	0.0059 (8)	0.0027 (8)	0.0042 (10)
C2	0.0466 (11)	0.0502 (11)	0.0542 (12)	0.0094 (8)	0.0066 (9)	0.0165 (10)
C3	0.0375 (10)	0.0618 (12)	0.0397 (10)	0.0139 (8)	0.0099 (7)	0.0146 (10)
C4	0.0333 (10)	0.0552 (11)	0.0349 (10)	0.0071 (7)	0.0105 (7)	0.0072 (9)
C5	0.0361 (10)	0.0496 (11)	0.0371 (10)	0.0041 (7)	0.0116 (7)	0.0072 (9)
C6	0.0456 (11)	0.0677 (14)	0.0384 (11)	0.0031 (9)	0.0054 (8)	0.0030 (10)
C7	0.0480 (12)	0.0649 (14)	0.0609 (14)	−0.0080 (9)	0.0089 (9)	−0.0032 (12)
C8	0.0537 (13)	0.0568 (13)	0.0692 (15)	−0.0040 (9)	0.0203 (10)	0.0128 (12)
C9	0.0479 (11)	0.0644 (13)	0.0487 (12)	0.0016 (9)	0.0140 (8)	0.0182 (11)
C10	0.0562 (15)	0.0813 (18)	0.0507 (14)	0.0172 (12)	0.0076 (10)	0.0254 (13)
C11	0.0506 (12)	0.0446 (11)	0.0432 (12)	−0.0029 (8)	−0.0034 (9)	0.0026 (9)
C12	0.0456 (11)	0.0517 (12)	0.0681 (14)	−0.0010 (8)	0.0162 (9)	0.0099 (11)
C13	0.0682 (13)	0.0445 (11)	0.0459 (12)	−0.0100 (9)	0.0213 (9)	0.0035 (9)
C14	0.0535 (12)	0.0425 (11)	0.0406 (11)	−0.0094 (8)	0.0038 (8)	0.0074 (9)
C15	0.0476 (11)	0.0448 (11)	0.0422 (11)	−0.0021 (8)	0.0064 (8)	0.0042 (9)
C16	0.0767 (15)	0.0604 (13)	0.0480 (13)	−0.0077 (11)	−0.0051 (10)	0.0151 (11)
C17	0.0659 (16)	0.0710 (16)	0.086 (2)	−0.0015 (12)	−0.0221 (13)	0.0247 (15)
C18	0.0536 (14)	0.0674 (16)	0.108 (2)	0.0078 (10)	0.0065 (13)	0.0165 (15)
C19	0.0631 (14)	0.0631 (14)	0.0649 (14)	0.0069 (10)	0.0185 (10)	0.0107 (11)
C20	0.118 (2)	0.087 (2)	0.0657 (18)	0.0003 (17)	0.0483 (16)	0.0180 (16)

### Geometric parameters (Å, º)

**Table:**

O1—C1	1.371 (2)	C10—H10C	0.97 (3)
O1—C11	1.401 (2)	C10—H10A	0.95 (2)
N1—C1	1.296 (2)	C10—H10B	0.97 (3)
N1—C5	1.376 (2)	C11—C12	1.386 (3)
N2—C11	1.285 (2)	C12—H12	0.9300
N2—C15	1.373 (2)	C13—C12	1.363 (3)
C2—C3	1.355 (3)	C13—C20	1.497 (3)
C2—C1	1.408 (2)	C14—C16	1.408 (3)
C2—H2	0.9300	C14—C15	1.409 (3)
C3—C10	1.496 (3)	C14—C13	1.426 (3)
C4—C6	1.407 (3)	C15—C19	1.404 (3)
C4—C3	1.427 (3)	C16—C17	1.354 (3)
C5—C9	1.398 (2)	C16—H16	0.9300
C5—C4	1.416 (2)	C17—H17	0.9300
C6—C7	1.360 (3)	C18—C17	1.391 (4)
C6—H6	0.9300	C18—H18	0.9300
C7—H7	0.9300	C19—C18	1.354 (3)
C8—C7	1.396 (3)	C19—H19	0.9300
C8—H8	0.9300	C20—H20A	0.956 (18)
C9—C8	1.365 (3)	C20—H20B	0.981 (18)
C9—H9	0.9300	C20—H20C	0.978 (19)
			
O1—C1—C2	114.47 (16)	C9—C8—H8	119.9
N1—C1—O1	119.50 (16)	C11—N2—C15	116.43 (16)
N1—C1—C2	126.03 (17)	C11—C12—H12	120.3
N1—C5—C9	118.13 (16)	C12—C11—O1	118.03 (18)
N1—C5—C4	122.58 (15)	C12—C13—C14	117.55 (18)
N2—C11—C12	126.19 (17)	C12—C13—C20	121.2 (2)
N2—C11—O1	115.61 (18)	C13—C12—C11	119.38 (18)
N2—C15—C19	117.56 (18)	C13—C12—H12	120.3
N2—C15—C14	122.48 (17)	C13—C20—H20A	113.8 (19)
C1—O1—C11	117.49 (14)	C13—C20—H20B	109 (2)
C1—N1—C5	116.00 (15)	C13—C20—H20C	111.1 (18)
C1—C2—H2	120.3	C14—C13—C20	121.2 (2)
C2—C3—C4	117.60 (16)	C14—C16—H16	119.5
C2—C3—C10	121.44 (19)	C15—C14—C13	117.96 (16)
C3—C2—C1	119.35 (17)	C15—C19—H19	119.9
C3—C2—H2	120.3	C16—C14—C15	117.77 (19)
C3—C10—H10A	110.7 (12)	C16—C14—C13	124.27 (19)
C3—C10—H10B	109.6 (13)	C16—C17—C18	120.8 (2)
C3—C10—H10C	111.3 (14)	C16—C17—H17	119.6
C4—C3—C10	120.92 (19)	C17—C16—C14	121.0 (2)
C4—C6—H6	119.5	C17—C16—H16	119.5
C5—C4—C3	118.42 (17)	C17—C18—H18	119.9
C5—C9—H9	119.6	C18—C17—H17	119.6
C6—C4—C5	118.45 (17)	C18—C19—C15	120.2 (2)
C6—C4—C3	123.13 (16)	C18—C19—H19	119.9
C6—C7—C8	120.43 (19)	C19—C15—C14	119.95 (17)
C6—C7—H7	119.8	C19—C18—C17	120.3 (2)
C7—C6—C4	120.92 (18)	C19—C18—H18	119.9
C7—C6—H6	119.5	H10A—C10—H10B	108.5 (18)
C7—C8—H8	119.9	H10C—C10—H10A	109.4 (17)
C8—C7—H7	119.8	H10C—C10—H10B	107 (2)
C8—C9—C5	120.81 (18)	H20A—C20—H20B	109.3 (18)
C8—C9—H9	119.6	H20A—C20—H20C	108.4 (19)
C9—C5—C4	119.28 (17)	H20B—C20—H20C	104.8 (17)
C9—C8—C7	120.11 (18)		
			
O1—C11—C12—C13	−174.70 (15)	C9—C5—C4—C3	−179.86 (14)
N1—C5—C4—C6	178.55 (15)	C9—C8—C7—C6	−0.9 (3)
N1—C5—C4—C3	−0.9 (3)	C11—O1—C1—N1	17.5 (3)
N1—C5—C9—C8	−178.88 (17)	C11—O1—C1—C2	−162.77 (16)
N2—C15—C19—C18	−179.87 (19)	C11—N2—C15—C19	−179.60 (17)
C1—O1—C11—N2	83.6 (2)	C11—N2—C15—C14	−0.5 (3)
C1—O1—C11—C12	−100.8 (2)	C13—C14—C15—C19	178.68 (16)
C1—N1—C5—C9	179.85 (15)	C13—C14—C15—N2	−0.4 (3)
C1—N1—C5—C4	0.9 (3)	C13—C14—C16—C17	−179.87 (19)
C1—C2—C3—C4	0.6 (3)	C14—C13—C12—C11	−1.2 (3)
C1—C2—C3—C10	−177.41 (18)	C14—C15—C19—C18	1.0 (3)
C3—C2—C1—N1	−0.7 (3)	C14—C16—C17—C18	1.4 (3)
C3—C2—C1—O1	179.64 (16)	C15—N2—C11—C12	0.6 (3)
C3—C4—C6—C7	179.46 (16)	C15—N2—C11—O1	175.70 (14)
C4—C5—C9—C8	0.1 (3)	C15—C14—C13—C12	1.2 (2)
C4—C6—C7—C8	0.6 (3)	C15—C14—C13—C20	−178.48 (19)
C5—C4—C3—C10	178.14 (17)	C15—C14—C16—C17	0.1 (3)
C5—N1—C1—O1	179.57 (16)	C15—C19—C18—C17	0.4 (3)
C5—N1—C1—C2	−0.1 (3)	C16—C14—C15—N2	179.68 (16)
C5—C4—C3—C2	0.1 (3)	C16—C14—C15—C19	−1.3 (3)
C5—C4—C6—C7	0.0 (3)	C16—C14—C13—C12	−178.83 (17)
C5—C9—C8—C7	0.5 (3)	C16—C14—C13—C20	1.4 (3)
C6—C4—C3—C2	−179.31 (16)	C19—C18—C17—C16	−1.6 (4)
C6—C4—C3—C10	−1.3 (3)	C20—C13—C12—C11	178.5 (2)
C9—C5—C4—C6	−0.4 (3)		
